# Automatic Annotations by Augmented Reality‐Enabled Laparoscopic Surgery

**DOI:** 10.1049/htl2.70031

**Published:** 2025-11-19

**Authors:** Alexander Winkler, Christian Heiliger, Thomas Heiliger, Ulrich Eck, Konrad Karcz, Nassir Navab

**Affiliations:** ^1^ Chair for Computer Aided Medical Procedures and Augmented Reality (CAMP) Technical University of Munich (TUM) Munich Germany; ^2^ Department of General, Visceral, and Transplantation Surgery Hospital of the Ludwig Maximilian University (LMU) Munich Germany

**Keywords:** augmented reality, data acquisition, endoscopes, surgery

## Abstract

Accurate labels of surgical procedures such as image segmentations or interaction labels are paramount for many of today's medical image computing tasks. Creating a dataset with these labels requires a great deal of manual work and relies on the involvement of medical experts, which is very time‐consuming and costly. We propose a pathway for the automatic generation of such labels utilizing the spatial and temporal registration between a patient, the anatomical model, tracked surgical instruments, and the surgeon's view of the patient. These requirements for the automatic generation of labels are identical to the requirements of many navigated and augmented reality (AR) enabled surgeries. The AR system, through 3D registration, has the defining ability to accurately overlay real objects with their virtual counterparts. Our approach collects the complete raw data (e.g. video, tracking data, calibrations etc.) that feeds a live laparoscopic AR system for later analysis. By converting these complete recordings of the surgery into different representations, the AR system generates valuable datasets as mere by‐products. Additionally, as our approach does not rely on visual input alone but on additional 3D information, the system can create labels even if the visual input is occluded or a tool interacts with tissue outside of the view of the laparoscopic camera. In this paper, we present a realization of this concept, then evolve this foundational idea into an interactive system that assists users in annotating surgical data. Finally, we gather and analyse feedback from six participants to evaluate the efficacy and user‐friendliness of our system.

## Graphical abstract

We present a method for the automatic generation of surgical annotations by leveraging the spatial and temporal registration inherent to augmented reality (AR)‐enabled surgery. Our system records and synchronizes complete multimodal data streams from live AR procedures to generate structured labels as by‐products of surgical navigation. A prototype implementation and user evaluation demonstrate the feasibility and usability of this concept.

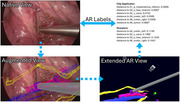

## 1 Introduction

Machine learning has transformed numerous fields, including healthcare and surgery. Particularly in surgery, it can assist in preoperative planning, intraoperative guidance, and postoperative assessment, improving precision, efficiency and patient outcomes. Supervised learning relies on carefully designed and annotated datasets. These methods learn from labelled data, where the correct output is provided during training. The quality, diversity and size of these datasets significantly influence the performance of the models. Many such datasets (e.g. [1, 2, 3]) are made available to other researchers, which greatly benefits the scientific community as creating them is labourious and time‐consuming [4].

Acquiring surgical videos is technically straightforward; But even though computer vision and machine learning are increasingly being applied to surgical videos, even fundamental tasks like object tracking remain highly challenging due to factors such as background clutter, occlusions, and illumination variations. Assigning high‐quality annotations that require surgical knowledge, such as in surgical workflow analysis, remains highly challenging, as they are often laboriously hand‐annotated by experts. [5, 6, 7]. For some tasks, such as pose estimation of instruments or anatomical deformations obtaining ground truth from real surgeries is even more difficult, due to the lack of direct measurement methods.

The comprehensive review on surgical data science (SDS) by Maier‐Hein et al. [8] among other aspects, identifies the following challenges regarding the acquisition of surgical data: (i) Not all data can currently be acquired, (ii) acquired data may not be permanently stored, (iii) stored data may not be structured, (iv) stored data may not be exchanged between systems, and (v) regulatory constraints make data acquisition, storage, and access challenging.

De Backer et al. [5] focus on manual and team‐assisted annotation strategies. They highlight the annotation burden in surgical AI and emphasize the need for hierarchical team structures and optimized frame‐sampling strategies to balance annotation detail with efficiency. While some annotation tasks can be delegated to novices, experts spend much time on producing clinically meaningful labels [9]. Therefore, automatically generating datasets or reducing the work by assisting a human annotator is highly desirable.

Augmented reality (AR) is an emerging technology that merges virtual and real environments, enhancing users' perception of their environment. Visualization can happen on a multitude of displays, be it head‐mounted displays, augmented monitors, projections etc. [10]. Medical AR does not merely superimpose data into users' perception of their environment but should increase knowledge and awareness. In a surgical setting, AR can provide surgeons with preoperative information, such as anatomical structures, plans, or navigation cues, directly in the surgeon's field of view [11]. The defining characteristic of AR is to create a perception for the user, that virtual and real objects share the same space. In visual AR this usually boils down to defining a spatial and temporal registration between the virtual and real content. Therefore, this technology provides a means to establish a link between preoperative patient data and the viewing modality in the operating room. To facilitate the co‐localization of digital and physical information, the AR system must create an internal representation (extended AR view) of the physical environment, which it perceives through sensors, such as cameras, tracking systems, and medical equipment. These sensor streams are then calibrated and synchronized so that the AR system creates outputs, in line with the surgical situation [11]. This internal representation, typically used to generate AR overlays in real‐time, contains rich, structured data about the procedure.

We observed that an AR system inherently performs much of the groundwork required for automatic annotation: Developing an AR system necessitates accurate spatial and semantic registration of the surgical environment, including instrument tracking, patient‐specific anatomy integration, and workflow‐aware visualization. By retaining and repurposing the internal representation, it can automatically be transformed into structured annotations for SDS applications. We directly go from AR scenes to annotations, without heavy postprocessing—the AR system has already done it. Crucially, AR does not inherently solve annotation tasks, but the same requirements for AR visualization make annotation a natural byproduct. Any researcher developing an AR system can, with minimal extra effort, produce high‐quality surgical datasets.

García‐Pereira et al. [12] propose a theoretical framework for AR annotations, defining them as virtual elements that provide contextualized information. They present a taxonomy that categorizes AR annotations based on four key dimensions: content, location, temporality, and interaction. The authors further emphasize the necessity of standardizing AR annotations across different systems to improve interoperability. Portalés et al. [6] describe annotation as an essential element of the mixed reality (MR) paradigm and present a system for adding virtual annotations, specifically bounding boxes, onto stereoscopic videos of robotic surgery for MR education applications. In their approach, a user selects a region of interest in one stereo view, which is then tracked over time using OpenCV‐based methods and matched in the other stereo view via block matching. Their focus is on producing annotated stereo videos for MR playback rather than on dataset creation for computational analysis.

Earlier works suggested acquiring data in AR and clustering images based on low‐level features, which the user can then annotate [13] for object recognition in an office environment. Sielhorst et al. [14] synchronize trajectories of similar movements between performances of novices and experts in an AR birth simulator using dynamic time warping (DTW) and calculate corresponding timestamps between them. Ahmadi et al. [15] also use DTW to synchronize workflow phases of cholecystectomies. As input, they use binary state vectors indicating which surgical instrument is used at which timestamp. By time‐warping to a labelled average surgery, they recover workflow phases reliably. Huaulmé et al. [16] had users perform surgical tasks in a virtual reality simulator and could perform low‐level physical activity annotations of a peg transfer task. On the other hand, image or point cloud annotation tasks may be done by users in virtual reality head‐mounted displays, which engages users through gamification [17]. HoloPointer [18] enables real‐time annotation on a laparoscopic monitor for intraoperative guidance. Instead of relying solely on verbal instructions or physical pointing, it allows the use of see‐through head‐mounted displays to control an interactive pointer overlaid onto the laparoscopic monitor with head gaze and share it with other users. This form of visual guidance, with less ambiguity than the currently used verbal guidance, led to more focused instrument movements. They conclude that their results make the AR pointer applicable for laparoscopic training.

Lecuyer et al. [4] propose an assisted annotation system for surgical video labelling, leveraging deep learning‐based pre‐recognition for identifying surgical phases and steps. They quantify the uncertainty of annotations by detecting errors and enforcing temporal consistency, improving annotation accuracy and efficiency. With data from medical robotics, forward kinematics, and 3D models, the instruments of a surgical robot can be projected into the image plane for pixel‐wise segmentation of the instruments. Unfortunately, this approach leads to inaccuracies, as accurate overlays in the image plane were never a goal of these systems. Pakhomov et al. automatically create inaccurate instrument segmentation and pose the problem as an unpaired image‐to‐image translation task [19].

Kitaguchi et al. [20] propose a deep learning‐based semantic segmentation approach for real‐time anatomical navigation in laparoscopic surgery. Their post‐hoc study highlights the feasibility of AI‐driven anatomical navigation in real‐time but acknowledges challenges in handling tissue deformation and occlusion. They discuss that landmarks of the anatomy could be extracted from preoperative CT scans, reconstructed as AR overlays, and integrated with their semantic segmentation model to create a more informative navigation system.

In summary, prior work has explored challenges in surgical video annotation, manual and assisted labelling strategies, and the use of AR for intraoperative guidance or visualization. While some studies explore AR for visual enhancement or interaction, they do not leverage the internal representations created by these systems for dataset generation.

This paper describes our AR Labels concept and how we captured and transformed the data required in AR‐enabled surgeries into representations useful for surgical data labelling tasks. We describe the concept that AR systems inherently contain the spatial and semantic understanding needed to generate rich annotations, and can therefore serve as a foundation for automated or semi‐automated dataset creation. Secondly, we describe a software that automates tasks of labelling AR‐enabled laparoscopic surgery. Third, we demonstrated a version of our software tuned to surgical triplet annotation of laparoscopic surgery to participants and summarized their feedback.

## 2 Methodology

**FIGURE 1:**
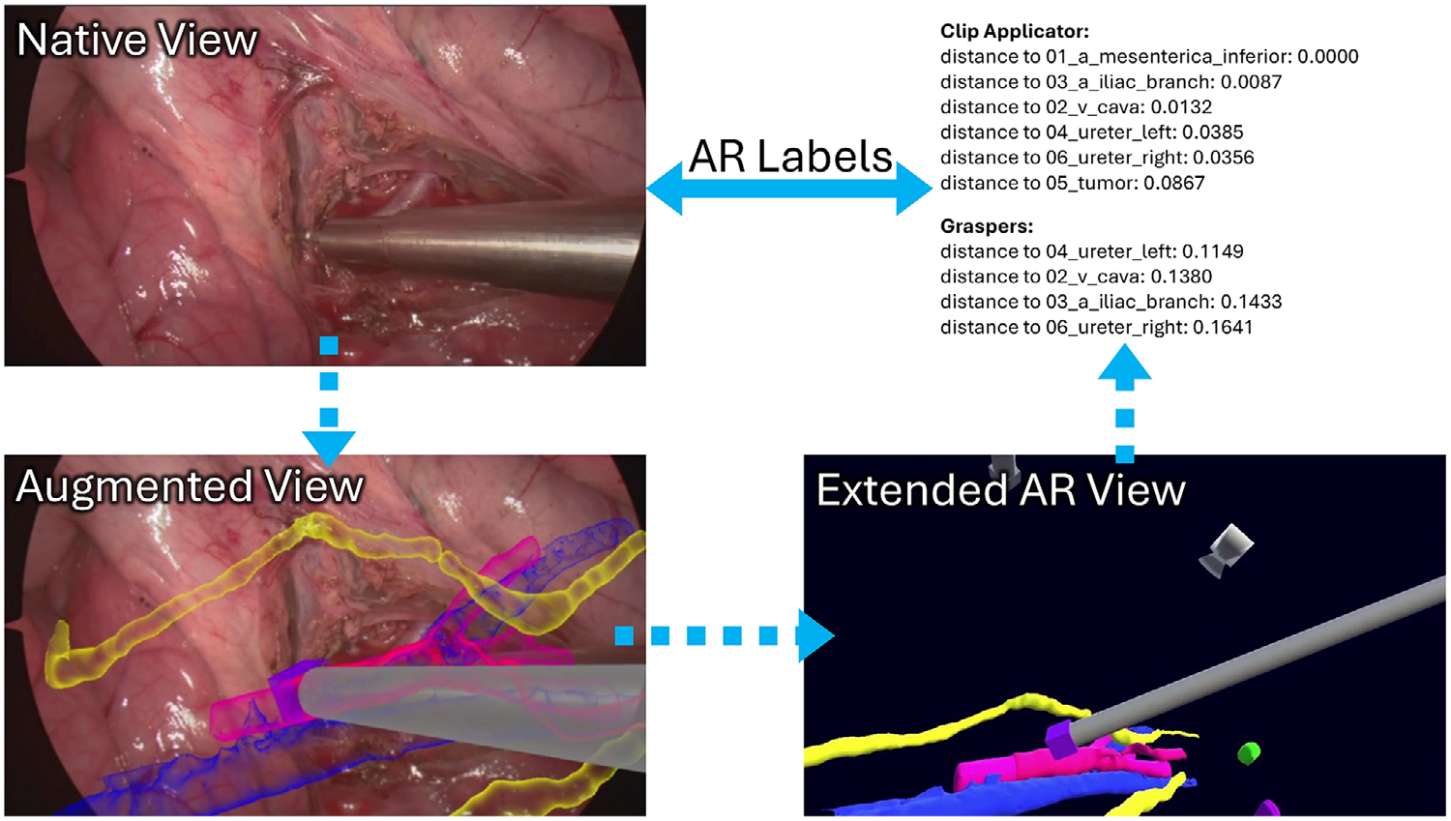
With AR Labels we connect an unaugmented native view to an augmented view. It has rich data attached, which we denote extended AR view facilitating a broader understanding of the scene. From it we derive rich labels of the scene, in this example distances between surgical instruments and anatomical structures. Ultimately, the user of AR Labels interacts only with the native view and the generated labels. Notably, this approach enables annotation of off‐screen instruments (e.g. graspers) and occluded structures (e.g. ureter), which would be difficult to label using traditional methods.

At the heart of AR Labels lies the understanding that we can pass semantic meaning from one modality to another, with AR being the connection. This is pictured in Figure 1.

Passing meaning between modalities is natural in the surgical workflow; for example if an anatomical feature is easier to see in an X‐ray, even though a specific surgery is performed under direct view, the surgeon today must mentally combine the preoperative information with the surgery site. Furthermore, we analysed a set of AR‐enabled surgeries and concluded that the requirements for good AR systems, which align virtual and real content closely and are well integrated with the surgical workflow, and automated labelling are very similar.

We propose that, beyond its immediate benefits for surgical procedures, AR is a powerful data‐collection tool. For AR in surgical workflows, detailed surgical data, including instrument positions, surgeon movements, patient anatomy, and procedural steps, must be captured within the context of the ongoing surgery. To render virtual representations of anatomical locations spatially correctly superimposed onto the laparoscopic video stream, the transformations of all tracked and calibrated objects must be known [10]. If the AR system successfully creates meaningful AR visualizations, its understanding of the surgical scene is good enough for the task at hand. The AR software was thus able to describe the surgical scenario in a very dense form, for example as the spatial relations between the objects. Suppose this data is not discarded after the visual output is displayed, but retained and correlated with complex labels. This data can be transformed into an invaluable source of rich data for data‐driven techniques, not only for AR‐enabled surgery but the corresponding non‐AR surgery as well.

### 2.1 AR‐Enabled Surgery

**FIGURE 2:**
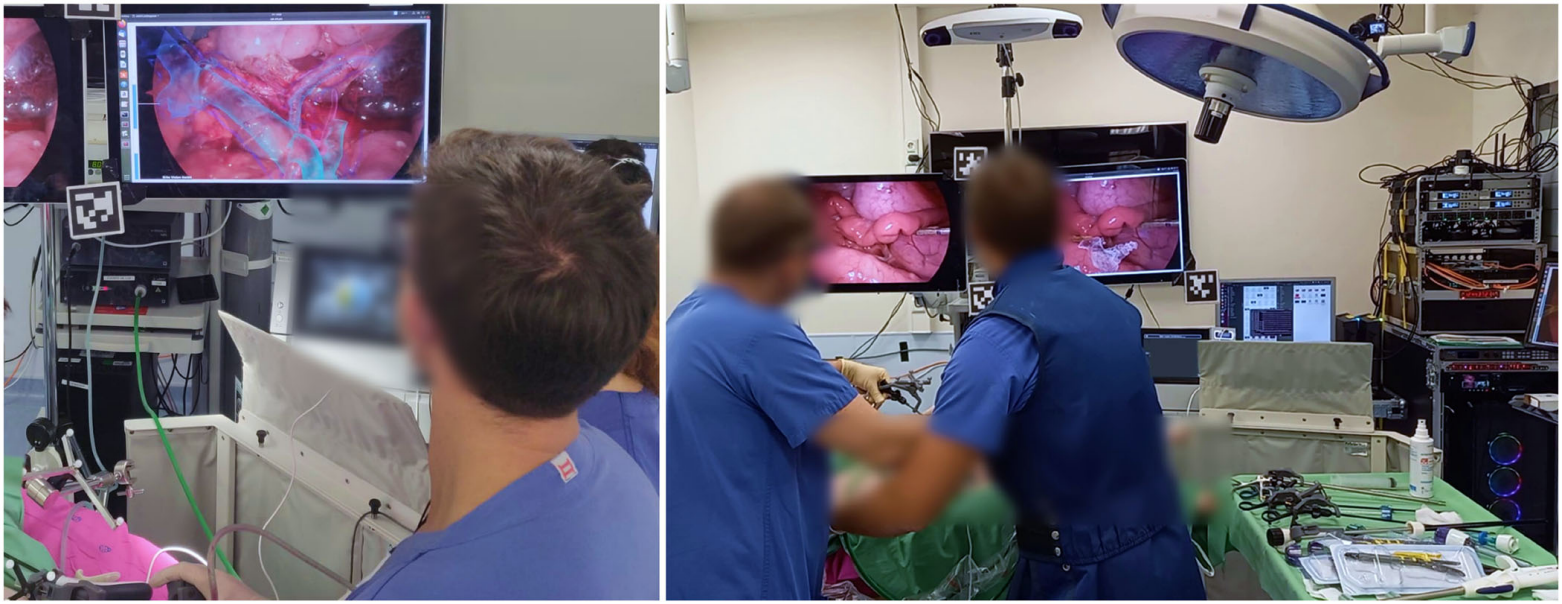
Our setup to perform and record laparoscopic AR‐enabled surgeries: (left) The output of the AR system is shown on an augmented monitor [10]. (right) An infrared tracking system detects passive tracking arrays attached to the laparoscope, instrument and patient to create accurate overlays of patient data on the laparoscopic video. Our recording hardware is seen in the right corner, consisting of a room camera, wireless microphones, a network connection to the AR system, a digital video recorder to record the laparoscopic video, and a PC, which stores tracking data, user interactions, and the status of the AR setup. For eye tracking performance analysis, we attached AR tags to the screens, but they are not part of our AR system.

Our intraoperative AR setup (Figure 2) consisted of an externally tracked 10 mm, 30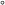 laparoscope and an infrared tracking system (Northern Digital Polaris Vega ST) with optical tracking arrays with retroreflective spheres, similar to [21, 22]. Individual tracking arrays tracked the laparoscope, patient table, and instruments. The registration between the targets and the respective objects was established through hand‐eye calibration for the laparoscope, 2D–3D intensity‐based registration with X‐ray images from a tracked mobile C‐arm to the CT scan for patient registration, and pivot calibrations for each instrument. Tracking arrays were attached not only to the laparoscope and surgical instruments, but also to the operating table, allowing the system to maintain a consistent coordinate frame despite any movement during the procedure. The quality of the laparoscopic camera calibration and hand–eye calibration was verified before each trial by visualizing landmarks on a tracked plastic phantom. The output of the AR system is shown on an augmented monitor [10], showing the laparoscopic video overlaid with virtual content on a standard operating room monitor. This AR setup combines the live video with preoperative imaging data, facilitating spatial alignment between the surgical field and the preoperative plan. Augmented content included 3D models of anatomical structures visualized with wireframe rendering or Fresnel‐derivative edge highlighting [23], instrument cutouts for correct occlusions between the virtual anatomy and the instruments [22], and semi‐transparently rendered spheres for target structures, overlaid in real time onto the live video. This setup allows the surgeon to view augmented information without wearing any special hardware, maintaining the conventional laparoscopic workflow.

18 preclinical trials were conducted on swine. All procedures received approval by the local ethical committee on animal experimentation.^1^ An on‐site radiologist manually segmented the desired structures in the CT scan with 3D Slicer^2^ [24].

The AR system could then show anatomies of interest, such as blood vessels, ureter etc., superimposed on the laparoscopic image with a target registration error of 8.2 mm and an end‐to‐end latency of 126 ms, which is acceptable for AR overlays in laparoscopic surgery [25, 26]. The registration accuracy was visually assessed by the lead surgeon using the aortic bifurcation as a key anatomical landmark, a well‐defined vascular branching point, comparing its appearance in the laparoscopic video to its AR overlay derived from the CT scan: The surgeon viewed the target structure from multiple angles and judged the alignment between the overlaid 3D model and the real anatomy. If the alignment was deemed insufficient, a technical operator adjusted the patient coordinate system offset interactively. This process was repeated iteratively until the surgeon confirmed the overlay was visually accurate, which we define as our reference alignment. For the actual surgical task and annotation generation, we reverted to the original, uncorrected registration produced by the automated system, as this reflects the realistic baseline performance that a deployable AR solution can achieve. This approach is aligned with observations in the literature: As noted by Malhotra et al. [27], current surgical AR systems rely primarily on visual assessment of alignment quality.

### 2.2 Recording

The objective of the preclinical trials aside from a usability study of the AR system, was to record the complete raw data of the input to the AR system, which resulted in an elaborate recording setup. FullHD video streams of the laparoscope, the AR system, and a room camera, as well as audio captured via lavalier and a room microphone were recorded on a digital video recorder (AJA Ki Pro). The tracking information of the infrared tracking system was forwarded by the AR application over network to a recording PC (running Ubuntu 18.04), which saved the tracking data, status information and user interactions into ROS (robot operating system^3^) bag files. To synchronize the data streams, the video recorder forwarded timecodes over MIDI, which were also stored in the ROS bags. Miscellaneous data, such as the CT scans and its segmentations, registrations, and calibrations were automatically copied from the AR system and stored on the file system of the recording PC. Thus, complementary pre‐ and intraoperative data were integrated into the dataset, such as CT scans both with and without capnoperitoneum, precise segmentations created by a radiologist, calibrations, and registration data created on the AR PC, as well as manual experiment phase annotations acquired via a tablet PC. This structure allows the time‐synchronized playback of all data streams, as they were created in the operating room, at a later time. Therefore, we can recreate the AR system's output, change visualization parameters, or transform the data into completely different representations. In summary, video, tracking, calibration, segmentations, registration outputs, and user interactions were streamed into the AR application, enabling frame‐by‐frame alignment of instruments and patient anatomy with the video. These synchronized data streams, required for intraoperative visualization, also formed the raw input for AR Labels. By capturing the same relevant data, the complete augmented scene could be reconstructed later without fidelity loss. This enabled synchronized replay, alternative visualizations, and label extraction without repeating the experiment.

### 2.3 Replay

For analysis and replaying the recorded data, a re‐implementation of the laparoscopic AR application in Unity^4^ was created, which allows to replay the data streams in a time‐synchronized manner. Loading the appropriate 3D models, registration and calibration files, placing them into an equivalent configuration in the scene hierarchy, and applying corresponding shaders to the virtual objects recreated the output of the AR system and let us replay the AR visualizations. Through the application of appropriate shaders to the 3D models of the different anatomies and instruments, the view of the virtual laparoscopic camera could be overlaid with renderings of the 3D models of the anatomy in several appearances, which correspond to different representations of datasets.

### 2.4 Replay Software as a Data Annotation Tool

**FIGURE 3:**
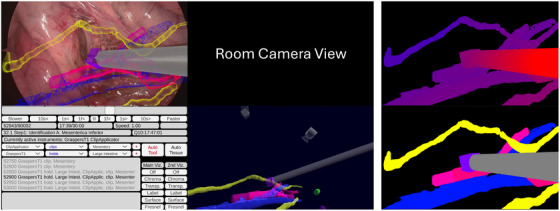
Visualizations in our annotation software: (top left) AR overlay over the laparoscopic video, next to the temporally synchronized room video (top centre, blacked out here due to privacy). (bottom left) The user interface offers transport controls (e.g. play, pause, timeline scrubbing) and allows assigning action triplets next to the freely adjustable extended AR view (bottom centre), visualizing the tracked structures. Notice how in the AR laparoscopic scene, only the stapler (purple tip) is visible, while in the extended AR view, the intestinal grasper (grey, at the top) can also be seen, which is just off‐screen in the AR view. Alternative visualization methods like pseudo chromadepth (top right) or segmentation masks created by rendering the virtual objects in solid colours (bottom right) can be chosen as well.

The replay software can not only recreate the original output of the AR system closely, but can also perform various measurements, display alternative visualizations, modify the output in arbitrary ways, or provide user interfaces for applications other than an intraoperative AR system. The replay of the recorded data allows for rendering the laparoscopic AR view with methods not intended for visualization but for storage in a dataset, such as label maps: The visualized 3D patient anatomy and surgical instruments of the AR system allow an implicit segmentation of the optical image by rendering the virtual anatomy with a fixed colour (see Figure 3 (bottom right)). The 3D instrument tracking also facilitates creating 2D masks for the instruments.

The built‐in physics engine can accurately and quickly calculate distances and collisions between instruments and the 3D anatomy. Through rule‐based methods relying on a closeness metric between instruments and tissues, a mapping between specific movements of the instruments relative to the anatomy, and a set of possible interactions for each instrument, an interaction can be derived.

The user interface allows for exploring the dataset interactively: The user can watch multiple time‐synchronized video streams (laparoscopic view or room video) from the procedure simultaneously. We provide a choice of different visualization methods of the overlays (physically based shading, semi‐transparency, pseudo chromadepth [28] (Figure 3 (top right)), Fresnel‐derivative outline highlighting [23] (Figure 3 (top left)) or label map (Figure 3 (bottom right)) and an additional freely positionable perspective on the 3D data (extended AR view, with the same choice of visualization methods, as seen in Figure 3 (bottom centre)). Transport controls (Figure 3 (bottom left)) allow to start/stop, fast‐forward, rewind or slow‐motion. Furthermore, based on the aforementioned rules, the system shows its current estimate of surgical action triplets, which the user can override from a dropdown menu (Figure 3 (bottom left)). Additionally, we show subtitles of the speech acquired through wireless microphones and the microphone of the room camera. The speech has been automatically transcribed through the locally‐running open‐source voice recognition software Whisper with the “small” model [29].

Our implementation runs in real‐time on a regular office PC (Intel Core i7‐6700K, 16GB RAM, NVIDIA GeForce GTX 1070) to replay the scene with any of the visualization techniques.

## 3 Case Study

We presented our implementation to several users, gave an in‐depth demonstration of our labelling software, and collected feedback in structured interviews.

The study was designed to evaluate the annotation workflow enabled by our AR Labels concept in a post‐operative setting, rather than the AR system's intraoperative guidance function. This allowed us to evaluate the practicality, efficiency, and clarity of using AR‐generated representations for annotation tasks. By focusing on the downstream annotation process, the study validates the central claim that AR‐based systems can reduce manual labelling effort in SDS workflows.

We interviewed six participants, three female and three male, $M_{\text{age}}=29.3$ years (${\text{SD}}_{\text{age}}=5.0$). Two were medical students, one assistant physician in gynaecology, one specialist in visceral surgery, and two engineers (computer science and mechanical engineering) in the medical field. Five had practical experience in medical annotation tasks, albeit most in annotating CTs or X‐rays. Two had previously annotated temporal data on surgical videos.

After consenting to the interview, we collected their demographics. Afterward, we demonstrated our annotation tool using the same consistent example surgery, explaining all functions in detail, followed by the system usability scale [30] questionnaire and a structured interview about their opinions on the software and the general concept.

Regarding the usability, the participants attested the system a score of $M_{\text{SUS}}=81.7$ (SD=6.5), which puts the system in a “very good” range. The participants appreciated that the software provides a complete understanding of the surgery, with multiple views on the procedure and different presentation modalities, giving a better overview of the actions of the surgical team. Most of them commented that the user interface in its current form was not very appealing and seemed cramped. They suggested making features optional, enlarging the laparoscopic view, and making the interface customizable. The AR view, superimposing renderings of the segmented structures over the laparoscopic video, was regarded as helpful by all participants, mainly stating that it makes identifying anatomies easier, and surgically less experienced people would greatly benefit from this information. One participant voiced concern that the virtual objects could occlude critical structures. Equally, all participants regarded the multiple views as beneficial. Most participants found the free‐moving 3D view helpful, especially for instruments outside the field of view of the laparoscope. At the same time, one participant did not understand the relationships of instruments and segmented structures in the 3D view.

They all regarded the automatic support in labelling as very useful, even if the suggestions by the system would sometimes be incorrect and need to be corrected manually. Some participants said they would experiment with it to understand which approach would save time. One participant was critical of the automatic tissue labelling feature, noting that our demonstrator always chose the tissue closest to the instrument tips.

Some participants would like to use the system on a tablet PC, while others regard mouse interactions as superior for these lengthy tasks. All but one participant dismissed using this concept in a virtual reality head‐mounted display. Two participants voiced privacy concerns, as the demonstrator included a room video in which the surgeons' faces were visible. Most participants confirm that they think that labels created with this system would be more accurate than relying on videos alone, but may take more time. All participants preferred using this tool over other annotation tools, which only support 2D videos and were enthusiastic about recommending this software to others for labelling tasks.

## 4 Discussion

Manual annotation of surgical videos is highly labor‐intensive and inherently limited by the sheer volume of data. For example, a 1‐h video at 30 fps results in over 100,000 frames, making frame‐by‐frame annotation infeasible in practice. As noted by De Backer et al. [5], most manual annotation strategies rely on sampling frames every few seconds to balance annotation effort with information coverage, at the cost of potentially missing important interactions or events. In contrast, AR Labels relying on tracking information can generate dense, frame‐level annotations automatically, including occluded anatomical structures or instruments that are outside the field of view, as shown in Figure 1 and 3. Unlike the method of Portalés et al. [6], which is limited by image‐based tracking challenges such as occlusions, lack of absolute depth calibration, and size inconsistencies between stereo pairs, our approach leverages the calibrated, spatially registered internal representation computed by an intraoperative AR system, encompassing instrument tracking and patient‐specific anatomy, so that temporally and spatially coherent annotations can be generated without additional visual tracking.

In our example scene, most of the time the grasper was out of frame. An expert might know that the grasper was holding the mesentery from the workflow and the visible distortion of the tissue in the frame. Our method also identified the interaction based on the 3D tracking data.

Although our implementation depends on the accuracy of the underlying registration and tracking systems, our approach establishes a scalable foundation for producing large and richly annotated datasets. We believe our approach can generate datasets that would be almost impossible to create with manual annotations from 2D videos alone, such as the hidden or out‐of‐view instruments or occluded anatomical structures in Figure 1.

While our concept can automatically generate rich annotations, our current implementation for detecting interactions or creating segmentations has limitations. This study focused on demonstrating the feasibility and usability of the AR Labels concept; we emphasize that the quality of labels produced by any implementation is inherently dependent on the underlying intraoperative AR system. First, factors such as registration method, overlay accuracy, clinical workflow, and the type of labels being generated (e.g. workflow annotations vs. pixel‐level segmentations) strongly influence label precision, and all labels are inherently affected by the registration accuracy of the AR system; spatial registration errors of several millimetres may lead to incorrect interaction labelling. Although systematic errors could potentially be corrected in a post hoc replay, for example by manually refining patient registration or incorporating anatomical deformation, such corrections were not applied in this work. Second, the current rule‐based interaction model is limited to spatial proximity, motion direction, and tool speed, which may result in incorrect interactions (e.g. when a tool rests on tissue without active engagement).

Consequently, AR Labels should not be considered a source of ground‐truth data, but rather as a powerful pre‐annotation framework that dramatically reduces manual workload and serves as a foundation for further refinement. The structured and multimodal nature of the data (combining tracking information, anatomical context, and aligned video) can simplify downstream tasks. For example, because the anatomy is already approximately registered to the video image and instruments are spatially tracked, offline models can operate with reduced complexity when refining labels, detecting instruments, or inferring tool–tissue interactions. Future work could enhance annotation quality through deformable registration, video‐based alignment corrections, or the incorporation of instrument state signals (e.g. activation of scissors or energy devices) to improve semantic precision.

Lessons learned from the recording aspects of our system emphasized the importance of design and planning: the replay system was challenging to implement as it was significantly different from the live system (e.g. data formats of the calibrations and different coordinate systems). While temporal consistency could be ultimately achieved, matching the timestamps of the different systems was unnecessarily complex, as different components of the recording setup stored data in different conventions. Careful considerations should be emphasized regarding data acquisition, storage, and access, to avoid blindly collecting excessive data.

A highly detailed analysis of the accuracy of our current specific implementation, subject to the limitations of our preclinical setup, would risk conflating these system‐specific factors with the broader capability of the concept. Nonetheless, we acknowledge the importance of quantifying label quality and variability, and in future work, we plan to include systematic comparisons with manual annotations, assessment of inter‐rater agreement, and explicit documentation of failure cases such as registration quality and drift or tracking loss. We also envision attaching automated quality indicators, such as overlay accuracy estimates or tracking confidence, to each label so that datasets can be filtered or weighted according to reliability. Thanks to our complete multimodal recording of each procedure, we are able to retrospectively analyse these metrics, an evaluation that would not have been possible without this comprehensive data capture. Ultimately, we aim to extend data collection to real human surgeries, enabling more realistic validation and comparison with automated methods that are tailored to human datasets, thus allowing for rigorous cross‐dataset and cross‐institution benchmarking of AR Labels.

## 5 Conclusions

We presented AR Labels, a way of transferring advances in one specific part of computer‐assisted interventions—AR‐enabled surgery—into the automatic generation of datasets. It leverages real‐time surgical AR systems to produce meaningful, registered annotations as byproducts. Unlike manually assigned AR annotations, our approach utilizes tracking data, intraoperative AR overlays, and instrument movements to generate structured annotations automatically. This not only reduces the workload of human annotators but also enables occlusion‐aware and multi‐modal annotations, addressing limitations of purely image‐based methods [6]. This can significantly accelerate the annotation process and reduce the manual workload, but human annotators remain essential to verify and refine the output. Similar to De Backer et al.'s hierarchical annotation [5], our approach provides high‐quality annotations while reducing expert intervention.

We observed that the requirements for generating automatic or semi‐automatic surgical annotations largely mirror those of an intraoperative AR system. At minimum, such a system must provide accurate spatial registration between anatomy, instruments, and the visualization modality; integrate patient‐specific preoperative data; and record synchronized multimodal streams for post hoc use. AR Labels does not require a flawless AR implementation; our own intraoperative system is not perfect, with overlay accuracy limited by externally tracked instruments and camera, as well as rigid patient registration. Nevertheless, in our user evaluation, participants considered it already valuable for automated labelling tasks. Label quality is ultimately tied to overlay accuracy, which depends on registration (including deformable), calibration, and tracking performance, all core aspects of high‐quality intraoperative AR.

We do not want to mislead anyone and make them believe that this approach can, on its own, fix the difficulties of navigated and AR‐enabled surgery. If the performance or accuracy of the AR system is poor, so will the resulting dataset. However, many navigated and AR‐enabled surgeries, existing or being developed, can create a lot more than visualizing patient data for only one surgeon. Therefore, AR systems should not be seen as ending their value at real‐time visualization, but the fused, optimized data they already generate is a rich and underused resource for downstream SDS tasks, to improve global surgical outcomes by enhancing data‐driven insights.

Taken together, we want to highlight the potential of AR Labels not only to improve annotation efficiency and completeness, but also to support the development of scalable, high‐quality datasets for SDS—unlocking applications that would be infeasible to pursue through manual video annotation alone.

We hope this concept inspires the community to rethink the value of the data already present in AR‐enabled surgical systems and to contribute to a shift in how we collect, interpret, and utilize surgical data for future innovations.

## Author Contributions

Alexander Winkler led the work on the AR Labels system, including its design and implementation, data acquisition, and execution of the user study. He also performed the primary data analysis and wrote the manuscript. Christian Heiliger contributed to the conception and planning of the preclinical experiments, performed surgical procedures, ensured compliance with the study protocol, provided surgical expertise for interpreting results, and assisted in manuscript preparation. Thomas Heiliger assisted in manuscript preparation and contributed to the literature review. Ulrich Eck designed and coordinated the data acquisition pipeline and provided technical feedback on the system architecture. Konrad Karcz contributed to the organization of the preclinical experiments, performed surgical procedures, and provided surgical feedback for system evaluation. Nassir Navab provided supervision of the technical and methodological aspects, contributed domain expertise in surgical data science, and supported the refinement and positioning of the manuscript.

## Funding

This work was supported by the German Federal Ministry of Education and Research (BMBF) under grant number 13GW0236B.

## Conflicts of Interest

The authors declare no conflicts of interest.

## Data Availability

The data that support the findings of this study are available on request from the corresponding author. The data are not publicly available due to privacy or ethical restrictions.

## Endnotes

1 ROB‐55.2‐2532.Vet02‐20‐212

2 Slicer 4.11,slicer.org

3 ROS Melodic, ros.org

4 Unity 2021.3.27f1, unity.com
